# Optimizing Care and Outcomes for People with Type 2 Diabetes – Lessons from a Translational Research Program on Insulin Initiation in General Practice

**DOI:** 10.3389/fmed.2014.00060

**Published:** 2015-01-29

**Authors:** John Furler, Irene Blackberry, Jo-Anne Manski-Nankervis, David O’Neal, James Best, Doris Young

**Affiliations:** ^1^General Practice and Primary Health Care Academic Centre, The University of Melbourne, Carlton, VIC, Australia; ^2^John Richards Initiative, School of Nursing and Midwifery, La Trobe University, Wodonga, VIC, Australia; ^3^Department of Medicine, St Vincent’s Hospital, The University of Melbourne, Fitzroy, VIC, Australia; ^4^Melbourne Medical School, The University of Melbourne, Parkville, VIC, Australia

**Keywords:** general practice, insulin, translational medical research, primary nursing, diabetes mellitus, type 2

## Abstract

**Background:** Clinical inertia, failure to intensify treatment according to evidence-based guidelines, leads to prolonged, avoidable hyperglycemia in people with type 2 diabetes (T2D). This is a challenge for General Practice and Primary Care, where most people with T2D receive most of their care. Sustained, integrated translational research programs are needed to embed effective treatments in routine practice, yet many challenges exist for developing such programs.

**Objectives:** To explore challenges and facilitators to implementing a translational research program focused on insulin initiation and titration among people with T2D in general practice and to identify key factors important to support and sustain such translation research in primary care.

**Operationalizing a program of translational work in primary care:** We describe a series of studies on insulin initiation and titration in general practice including theory and qualitative work (Phase 1), a small feasibility and acceptability pilot (Phase 2), a large scale pilot (Phase 3), and a pragmatic cluster randomized trial currently under way (Phase 4). We used mixed methods to explore practice level implementation issues, and reflective investigator discussions to explore broader research program sustainability.

**Challenges for translational research in primary care:** Key facilitators and barriers at practice and research program levels, include: Appropriate funding structures to secure long-term capacity building and people support; Building and maintaining linkages between communities of practice, primary and secondary/tertiary care researchers, institutions, and industry partners; Strategies for engagement and support for practitioners and participants.

**Conclusion:** Building effective and sustainable translational research programs are critical for developing evidence-based policy that drives improved outcomes at a population level. Diverse sources of funding that support extensive and sustained trans-mural collaboration as well as engagement with practitioners, patients, and policymakers in the field are crucial.

## Introduction

The rising global epidemic of type 2 diabetes (T2D) places primary care at the frontier of an effective health-care response. Implementation of evidence-based treatments in primary care, where most patients receive almost all of their diabetes care, can for example help patients achieve glycemic targets early in their illness, which is important for improving long-term patient outcomes. Yet, most people in the community with T2D continue to have glycemic levels out of target.

Insulin is an evidence-based treatment for achieving normoglycemia in T2D. Use of long acting insulin analogs with patient-driven algorithms is feasible, safe, effective (1, 2), and associated with improved patient satisfaction (3). Early use of insulin for people with T2D is supported by international guidelines (4). Yet, starting insulin is often delayed in clinical practice, particularly in general practice leading to prolonged hyperglycemic burden for patients (5).

This is an example of a “translational gap.” Bridging such gaps to improve research impact in the real world of clinical care is a global issue. It is the focus of Clinical Translation Science Centres in the US (6), the National Institute of Health Research in the UK (7), and the Research Translation Faculty in the National Health and Medical Research Council (MRC) in Australia. All these initiatives aim to bridge the “valley of death” between research evidence and health policy and clinical practice.

The “T3 translational gap” covers “Translation to Practice” and is focused on ways to disseminate and implement recommendations from clinical efficacy studies into general clinical practice. T3 translational research is particularly important for primary care, where the “grand challenge” (8) is to design studies that account for primary care’s unique context, the uncertainty and the generalist approach that are at the heart of primary care practice.

There is now a growing science of implementation (9, 10). The MRC framework for the development and implementation of complex health services interventions supports a stepwise approach, based on theory to guide the development of clinically relevant interventions and their implementation in real-world practice (11).

There are numerous reports of individual translational studies, but few descriptions of sustained programs of linked translational studies. We describe a program addressing the translational gap around insulin initiation in general practice for people with T2D who have glycemic levels out of target. The details of each of the component studies have been reported elsewhere. Our aim in this paper is to illustrate the use of the MRC framework in operationalizing a sustained program of T3 translational research in primary care. Our second aim is to identify factors important to supporting and sustaining such translational research in this setting.

## Our Program of Work

### Rethinking insulin initiation: the context for our work

It makes sense to base insulin initiation for people with T2D in routine general practice. Metabolic control in diabetes is often just one facet of managing multiple co-morbidities, so integrating a patient’s diabetes care with care for other conditions seems important. This could also reduce costs and be more acceptable to patients.

The reasons that many people with T2D do not have glucose levels within target are complex. Patients and clinicians struggle with the complex, progressive nature of this condition. Constant monitoring, adjustment and intensification of lifestyle, and medications are needed. For clinician and patient, the challenges associated with caring for and living with such a complex and dynamic chronic condition (12) may underlie practitioner “clinical inertia” (13). Delayed initiation of insulin may also stem from patients “psychological insulin resistance” (14). Health system and organizational factors are also important. For example, people with T2D are commonly referred to specialist care for insulin initiation (15, 16). Where general practice acts as a “gatekeeper,” difficulties accessing endocrinologists and diabetes nurse educators (DNEs) due to cost and limited availability can cause delays. Intermediate care, outreach clinics, and special interest practitioners (17) have all been suggested as ways to address this. Yet, gaps in treatment and achievement of targets persist.

**Figure 1:**
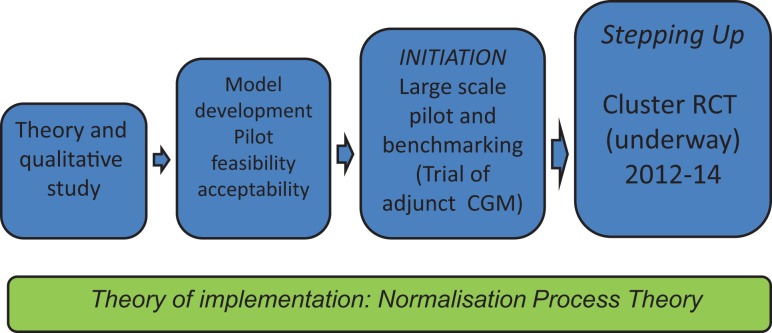
**Program of work and underpinning theory**.

This complex clinical problem is the focus of our research program (Figure 1). Consistent with the MRC framework, we began with an exploratory qualitative study (18), leading to the development of a practice-based intervention tested for feasibility and acceptability (19), before undertaking a larger pilot (20) leading to a cluster randomized controlled trial (RCT) (21), the final outcomes for which are expected in mid-2015.

Each study included its own evaluation, appropriate to the level of the study. Each study was approved by the Human Research Ethics Committee of Melbourne University. A core group of investigators, including a patient representative has been involved in each of the studies, with additional investigators added as the size and complexity of the studies increased.

### Theoretical basis for a translational research program

Rather than using psychological theories of behavioral change, we based our work on normalization process theory (NPT) (see Figure 1), a sociological theory of implementation, which describes how new practices become incorporated into routine clinical care as a result of individual and collective work (9). NPT describes how, a new practice becomes “normalized” as the people involved make sense of it, engage with and invest in it as they work, understand and agree who is involved in the new practice, and reflect on how the work is going.

Normalization process theory suggested that developing our research program would need patient representation as well as all members of the multidisciplinary diabetes team, including endocrinologist, DNE, general practitioners (GP), and generalist practice nurses (PNs). In Australia, most practices employ at least one PN (22), and they play an important role in chronic condition care, although not to the extent seen in countries such as the UK. All of these groups are involved in the “work” of insulin initiation.

## Operationalizing a Translational Program of Research in General Practice

### Study 1: Understanding the work involved in insulin initiation in general practice

A qualitative study (14) explored GPs and DNEs stories of “success” and “failure” in initiating insulin from their own practice while patient interviews used an illness narrative approach (23). This study captured the social, inter-professional, and organizational scaffolding of diabetes work that surrounded this key therapeutic moment. We identified a contrast in how people understood the aim of diabetes care in general practice (caring for disease versus caring for person), which shaped their perception of insulin initiation. While we identified uncertainty about who was seen as responsible for starting insulin, patients valued any professional willing to work on issues the patient themselves considered important.

Our findings suggested that a key challenge lay in supporting the integration of the technical work of insulin initiation with the generalist work structures and relational ethos of general practice through:

1. A clear and workable, in-practice system for referral and delegation of clinical work to integrate insulin initiation within the busy, reactive, time-pressured day-to-day GP clinical work;
2. Clarifying and acknowledging the aims and objectives of diabetes care from both the patient and clinician perspective; and
3. Clarifying the roles of GP, PN, DNE, endocrinologist, and patient in initiating insulin.

### Study 2: A model of care with an enhanced role for the practice nurse – the “Stepping Up Program”

Based on Study 1, we developed an intervention model of care explicitly incorporating an enhanced role in insulin initiation for the generalist PN (19). This enhanced role acknowledged the busy workflow of day-to-day practice and was designed such that GPs could delegate an active role for the PN to introduce and discuss the idea of insulin initiation with patients. While GPs retained prescribing authority, our model of care was based on the existing relationship between patients and GP–PN teams and allowed the possibility for in-house commencement of insulin, avoiding the need for referral and associated delays. Our intervention incorporated practitioner education [using simple, understandable evidence-based tools and algorithms for basal glargine insulin initiation and titration (1)], in-practice system change and minimal phone-based mentoring/support for the PN from a DNE and endocrinologist if needed.

The training and intervention resources were pragmatic, having been developed by GP and other primary care researchers. They acknowledged the multiple competing priorities in general practice and the need to tailor management to the individual patient and provider circumstances.

After a pilot of this model of care in five practices, qualitative evaluation involved workshops, focus groups, and interviews for clinicians and patients. Sustained use of the model in practice beyond the pilot suggested acceptability and generalizability. We found examples of how features of generalist practice were mobilized to the technical task of initiating insulin. For example, continuity of therapeutic relationships over time, biographical knowledge of patients, and whole person care were used proactively within the work of insulin initiation. Patients were able in this way to engage with the work of starting insulin in their own time and on their own terms. GPs and PNs saw this as coherent and meaningful work and that the model of care, tools, and resources supported “collective action” to address this gap in practice.

Challenges we identified included the need to continuously negotiate clinical autonomy at a local practice level, based on a shared understanding of scope of practice for PNs. There was concern about the lack of structural financing for the PN time involved in this work if it were to be scaled up at a practice level.

### Study 3: Field testing the model of care more widely

A larger pilot (20) in collaboration industry partners involved a non-randomized study of the model of care in 22 practices and 92 patients. We nested a study of retrospective continuous glucose monitoring (rCGM) within this pilot. Evaluation using a before and after design found a significant fall in HbA1c (24).

This study identified an evolution in the nature of the intervention model of care. The need for more intensive study DNE involvement with PNs and patients because of the embedded rCGM study partly changed the intervention. The model of care now included more DNE support for PNs on a face-to-face basis, rather than minimal phone support. We also incorporated more structured titration tools, including the addition of glulisine (short acting) insulin at one meal time if needed.

### Study 4: Evaluating the outcomes of the model of care

Our current cluster RCT (21) involves 75 practice and 266 patients, randomized at the practice level to receive the model of care intervention or usual general practice care. Follow-up data collection will be completed by March 2015.

This trial has allowed flexibility and a tailored approach in the implementation of the intervention, particularly relating to the level of DNE support. The study DNE has spent significant face-to-face time supporting PNs in some practices to embed and normalize the intervention, while other practices have required minimal support post-training. Evaluation has included interviews with GPs and PNs, which will be extended to patients as the trial is completed.

## Challenges for Translational Research in Primary Care

Changing clinical practice involves understanding and responding to the complex social and clinical milieu within which GPs, practice staff, and patients work. This has required a sustained and iterative process of development, implementation, and evaluation guided by the MRC framework.

### Using the MRC framework as guidance in designing, implementing, and sustaining our intervention

#### Addressing perceived needs of patients and providers

One important issue in implementing a new complex practice is “fit” with the perceived needs of those practitioners and patients who are expected to take up the new practice. This is the “sense making” domain of NPT. One way to try to match an intervention to the needs and preferences of end users is to explicitly incorporate options and preferences as part of an intervention (25). Our method of trying to achieve “fit” with the needs of end users was to use the MRC framework to guide an iterative process of consultation and intervention adaptation in our overall program of work, similar to the way Barley and colleagues describe the development of their intervention for depression in patients with heart disease (26). In addition, while our model of care intervention did not explicitly include patient preference and options as a formal element of the intervention, we assumed that patient preferences would be active elements within the routine clinical care provided to them. In other words, we aimed to engage patients for whom insulin was an appropriate clinical option for GPs to consider, and to provide GPs and PNs with the skills, and confidence and systems in their practice to discuss this with patients and to act on this if the patient decided to give that treatment option a try. In fact, a number of patients in our pilot studies and in our current RCT chose not to start insulin.

#### Addressing inter-professional tensions

One element of meeting the needs of users lay in addressing inter-professional tensions as the program of work evolved. This tension, which emerged in our early qualitative work, centered on perceptions that the professional expertise and scope of practice of DNEs was being challenged by the new model for insulin initiation in general practice (27). This became evident in encounters between the investigator and research staff (with backgrounds as GP, DNE, endocrinologist, and PN) and clinical DNEs in the field, at a range of meetings where both PNs and DNEs were attending and at which our work was discussed.

Addressing this required a sustained communication of the ethos and nature of our intervention and building genuine inter-professional bridges. This included presentations at conferences and meetings giving opportunity for stakeholders to engage and identify goals we held in common. Critical to this dialog, and consistent with a key T3 translational principle (28), has been ensuring an investigator and research team composed of a patient representative and active “clinician researchers” that included all of the key professional groups and able to speak credibly with clinicians in a range of settings.

#### An adaptable and flexible intervention

The MRC framework notes that the framework stages may “… not follow a linear or even a cyclical sequence” (11). Chambers et al. (10) highlight that an intervention is never finally optimized but rather continuously in development and is potentially enhanced and strengthened while being implemented in real-world practice. If a research team is open and able to accommodate learnings from the field in iterative cycles of design, implementation, and evaluation, this can mean an intervention can evolve, approaching greater coherence and fit (9) with routine practice in the field.

This was a helpful guidance for our work, where the amount of DNE face-to-face support varied over the studies to achieve a flexible hybrid in the current study where this is individualized and tailored to the GP and PN needs. This is consistent with the way the model of care would likely work in a post-trial state, where a central organization provided DNE consultancy services that varied in reach and uptake, in part determined by practice need. This would require careful monitoring to identify unmet need. We found that some practices used our flexible delivery approach to delegate routine diabetes care to the study DNE. Some degree of negotiation from the study DNE was needed to ensure appropriate support and engagement was achieved.

#### Dove-tailing research with day-to-day general practice work

The guidance provided by the MRC framework helped us to evolve our intervention to overlay the busy work of day-to-day general practice as a “minimally disruptive” intervention. We wanted to place few demands on practices in recruitment and data collection while allowing them to set their level of their engagement with an intervention increasingly adapted to the realities of practice. Continuous engagement with practices, some of whom have continued involvement from study to study, has been built through personal relationships, retaining clinically trained research staff and use of regular newsletters and communications with our community of clinicians and patients. This needs significant resources and staff continuity.

### Supporting and sustaining T3 translation research in primary care

Sustaining a coherent and cohesive research team, with junior and senior clinician researchers, from primary and secondary care, over a period time to allow the development of a research idea through the stages of the MRC framework has been an important base for this work.

In UK, this is recognized as a priority in the calls for “… funding opportunities …encouraging interdisciplinary working; … opportunities at the interface of medicine, public health and social care; … supporting emerging research groups, new models and methodologies” (11). In our case, without a commitment from funding bodies to support the stepwise approach of the MRC framework, we needed to opportunistically source funds from mainstream research councils, government tenders, NGO and professional groups, and industry sources to support people and field work.

This can mean stretching and diverting the focus of work to fit with research priorities in currently available funding streams, an opportunity cost that can threaten research intensity and quality and staff continuity. Our experience is illustrative, supporting the MRC call for “greater investment in developmental studies prior to large scale evaluations, and in implementation research [which] will help to ensure a better return on investment in evaluation studies” (11).

## Conclusion

Sustaining a linked program of translational research in general practice and primary care is challenging work. It is challenging to researchers as their theories and interventions struggle to match the complexities of practice. It is challenging to funders to recognize the way flexibility, innovation, and co-creation involving patients and practitioners are necessary to generate research that is relevant, to optimize interventions, and to really support changed practice across a range of settings and participants (10, 29). The challenge for primary care practitioners and researchers is to advocate and argue for the funding and support structures to foster such open innovation.

## Conflict of Interest Statement

The authors declare that the research was conducted in the absence of any commercial or financial relationships that could be construed as a potential conflict of interest.
